# Knowledge, Attitudes, and Practices Regarding Ebola Virus Disease Among Medical Students and Their Implications for Outbreak Preparedness

**DOI:** 10.7759/cureus.110595

**Published:** 2026-06-10

**Authors:** Rania A El-Kady, Abdulelah F Alasmari, Sanhat S Alotaibi, Abdullah T Rawa

**Affiliations:** 1 Medical Microbiology and Immunology, Faculty of Medicine, Mansoura University, Mansoura, EGY; 2 Pathological Sciences, Fakeeh College for Medical Sciences, Jeddah, SAU; 3 College of Medicine (MBBS Program), Fakeeh College for Medical Sciences, Jeddah, SAU

**Keywords:** ebola virus disease, emerging, infectious disease, kap questionnaire, preparedness, saudi

## Abstract

Introduction

Ebola virus disease (EVD) is a life-threatening illness caused by the Ebola virus (EBOV), a member of the *Filoviridae* family. In this study, we aimed to investigate the knowledge, attitudes, and practices of medical students at Fakeeh College for Medical Sciences, Jeddah, Saudi Arabia, concerning Ebola virus disease.

Methods

We conducted a cross-sectional study from January 2022 to June 2022, involving 198 students. We created a structured self-administered questionnaire to collect data from the participants. The questionnaire was disseminated to eligible students via official WhatsApp (Meta Platforms, Inc., Menlo Park, CA, USA) groups.

Results

The 198 respondents had a mean age of 21.87 ± 1.26 years, and 53% (n = 105) were female students. Third-year medical students were the largest cohort (42.4%; n = 84), followed by second-year (34.9%; n = 69), and fourth-year students (22.7%; n = 45). The participants had a poor knowledge score overall, with only 16.7% (n = 33) answering 70% of the knowledge questions correctly. The attitudes of the students toward EVD survivors were mixed. In terms of practical competence, most students did not attend any workshops on how to manage Ebola cases (79.3%; n = 157). Furthermore, only 25.7% (n = 51) knew the importance of donning gowns and gloves to protect healthcare workers against EVD.

Conclusions

Our study not only provides insights into how medical students understand and feel regarding EVD, but also highlights notable gaps in knowledge and practical readiness. Educational programs focusing on transmission dynamics and management of EVD are therefore highly recommended. Additionally, more hands-on training is essential to increase the preparedness of future healthcare workers to face potential Ebola outbreaks with confidence and ability.

## Introduction

Ebola virus disease (EVD), previously Ebola hemorrhagic fever, is a deadly zoonotic viral infection of humans and other primates. It was originally reported in 1976 following two concurrent outbreaks in Southern Sudan and the Democratic Republic of the Congo near the Ebola River, after which the virus was named [1]. EVD has a considerable case fatality rate ranging from 51.6% to 69.4% (average 60.6%), making it one of the most lethal viral infections in the last few decades [2].

Ebola virus (EBOV), the etiologic agent of EVD, is a member of the *Filoviridae* family, genus *Ebolavirus*. The virion is filamentous in shape and contains a single-stranded, nonsegmented, negative-sense RNA genome. In addition, the viral nucleocapsid is surrounded by a lipid envelope acquired from the plasma membrane of the infected host cells [3]. The EBOV genome displays a remarkable genetic heterogeneity because of its error-prone RNA polymerase, with diverse viral mutants having been isolated from different epidemics [4]. To date, the genus *Ebolavirus *includes six distinct strains: Zaire ebolavirus, Sudan ebolavirus, Tai Forest ebolavirus, Bundibugyo ebolavirus, Bombali ebolavirus, and Reston ebolavirus [5].

Transmission of EBOV occurs through unprotected physical contact of broken skin and mucous membranes with bodily fluids of infected animals or humans, including blood, saliva, sweat, tears, urine, feces, vomitus, breast milk, and semen. Furthermore, the virus can spread through indirect contact with contaminated surfaces and fomites, including non-sterile medical supplies [6]. Despite being rare, EBOV dissemination by sexual exposure to a convalescent or survivor of EVD is feasible, as the virus remains in semen for about six months following the acute phase of the disease [7].

The usual incubation period of EVD is 2-21 days (average 8-10 days). In the early stage of the disease, patients experience an abrupt onset of fever, chills, headache, sore throat, muscle aches, and fatigue, followed by anorexia, nausea, vomiting, diarrhea, and abdominal pain [8]. As the condition worsens, patients may experience rash and hemorrhage from mucous membranes and skin. Severe cases may be complicated with hypotension, shock, and multi-organ dysfunction [9]. Moreover, some survivors may suffer from lingering musculoskeletal, ophthalmologic, and neurologic sequelae, collectively referred to as post-Ebola syndrome (PES) [10].

A primary responsibility of healthcare workers (HCWs), including future clinicians, is to curtail outbreaks of emerging and re-emerging infectious agents. Although no local Ebola transmission has been reported in Saudi Arabia, recent Saudi data on healthcare infection-control preparedness highlight the need for sustained readiness for emerging infectious threats [11]. To mitigate the risk of transmission and respond effectively during potential Ebola outbreaks, healthcare practitioners should have in-depth knowledge of the disease. In this cross-sectional study, the primary objective was to assess what is known (knowledge), believed (attitude), and done (practiced) by the medical students at our institution with respect to EVD. The secondary objectives were to identify predictors of good knowledge of EVD and explore the correlations between KAP (Knowledge, Attitudes, and Practices) domains.

## Materials and methods

Ethical considerations and informed consent

The study design was reviewed and approved by the Institutional Review Board (IRB), Dr. Soliman Fakeeh Hospital, Jeddah, Saudi Arabia (decision no. 140/IRB/2020, dated November 5, 2020). Prior to data collection, participants were informed about the study purpose, which was briefly outlined in the introduction section of the e-questionnaire. They were assured that their responses would remain confidential and that their anonymity would be maintained throughout the study. In addition, they were informed that their participation in the questionnaire was voluntary and that they had the right to withdraw from the study at any time without any negative consequences. After reading an informed e-consent, students were directed to the e-questionnaire. The informed consent approach was also reviewed and approved by our IRB to ensure adherence to ethical guidelines.

Study design

We conducted this student-based, descriptive, cross-sectional study at Fakeeh College for Medical Sciences (FCMS), Jeddah, Saudi Arabia, from January 1, 2022, to June 30, 2022. FCMS provides four undergraduate programs: Bachelor of Medicine, Bachelor of Surgery (MBBS), Bachelor of Science in Nursing (BSc Nursing), Bachelor of Science Medical Laboratory Sciences (BSc MLS), and Bachelor of Doctor of Pharmacy (Pharm-D). The recruitment was limited to MBBS students in their second, third, and fourth years to ensure a homogenous and academically comparable cohort and increase the reliability and validity of our findings. Students were eligible if they were enrolled full-time and provided informed consent, while those who had postponed a semester, withdrawn, or declined to take part in the study were excluded.

Sample size

We calculated the sample size at 5% level of significance and 80% power of the study, using the following formula [12]:

$N = \frac{Z^{2} \times P \times (1-P)}{d^{2}}$

where N = minimum sample size; Z = 1.96 for a 95% confidence level; P is the proportion of the desired attribute, and equals 0.88, representing the proportion of students who have good knowledge of EVD from previous literature [13]; and d is the degree of accuracy (margin of error) and equals 0.05. The minimum sample size calculated according to this formula is approximately 163 students. A total of 198 medical students were finally included in the study.

Study tool

To assess medical students’ KAP regarding EVD, we created a structured self-administered questionnaire after an extensive review of the literature. The questionnaire consisted of 33 questions and was divided into four sections: (a) demographic characteristics, (b) knowledge of EVD, (c) attitudes toward EVD, and (d) practices related to EVD. The questionnaire underwent expert review by three specialists in Medical Microbiology and Public Health to evaluate the relevance, clarity, and comprehensiveness of the items, thereby establishing content validity. Their feedback was used to refine the questionnaire, ensuring that it effectively captured the KAP of participants regarding EVD. In addition, the questionnaire was pilot-tested among a group of 50 volunteer students to assess its clarity before full-scale distribution, providing evidence of validity. The internal consistency reliability of the questionnaire was evaluated using Cronbach’s alpha, yielding coefficients of 0.828, 0.788, and 0.815 for the KAP domains, respectively. Overall, the three domains demonstrated satisfactory psychometric properties.

Data collection

The questionnaire (see Appendices) was circulated among eligible students during the second and third semesters (between January and the end of June 2022). It was created using a secure Google form and disseminated through the students’ official WhatsApp (Meta Platforms, Inc., Menlo Park, CA, USA) groups. To ensure data integrity, the students were instructed to submit their answers only once to avoid duplicate responses and given reminders every week to complete the questionnaire.

Scoring

To measure the extent of knowledge, 12 questions were used. One point was awarded to each correct answer, and 0 to incorrect answers. The knowledge score was determined by adding up the correct responses in each section. Participants who answered ≥70% of the knowledge questions correctly were considered to have good knowledge. For attitude, 10 questions were used. A scoring system was applied, in which 1 point was given for a positive attitude toward EVD, and no points were given for negative attitudes. Scores ≥70% were deemed to reflect positive attitudes. Seven questions were used to assess practice, and participants scoring ≥70% were considered to have demonstrated good practice. Using a 70% cutoff facilitates comparison with other studies that have employed the same threshold, thereby enhancing consistency in the reporting and interpretation of findings across the literature. This cutoff is also commonly seen as a practical midpoint, as it is sufficiently high to differentiate adequate from inadequate knowledge, attitudes, or practices, while remaining realistically achievable for most participants [14].

Statistical analysis

All data were entered and analyzed using IBM SPSS Statistics for Windows, version 26.0 (IBM Corp., Armonk, NY, USA). Continuous variables were described as means ± standard deviation. Independent samples t-tests were conducted to compare the means of two independent groups. Categorical variables were presented as numbers and percentages, with Pearson’s chi-square test used for comparison. Odds ratios (ORs) and 95% confidence intervals (CIs) were calculated to determine the strength of any association. A one-way analysis of variance (ANOVA) was performed to assess score differences between students according to their academic year. Univariate and multivariate logistic regression analyses were performed to identify the contributing factors and independent predictors of good knowledge of EVD. To examine the relationships between knowledge, attitudes, and practices domains, Pearson’s correlation coefficients (r) were calculated. The significance was set at <0.05 for all analyses.

## Results

A total of 198 students participated voluntarily in the study by completing the questionnaire. The cumulative response rate was 44% (n = 87). The mean age was 21.87 ± 1.26 (range 20-24 years), with 53% (n = 105) of the respondents being female students. Most of the respondents were Saudi nationals (85.4%; n = 169). Third-year medical students were the largest cohort (42.4%; n = 84), followed by second (34.9%; n = 69) and fourth-year students (22.7%; n = 45). The internet served as the predominant source of information about EVD (51.5%; n = 102), followed by lectures (18.7%; n = 37) and television (13%; n = 26), whereas newspapers were the least common source (7.1%; n = 14).

Knowledge of EVD among medical students

Table 1 summarizes the response of recruited students to the knowledge-related questions. Of the 198 students, approximately 43% (n = 86) were aware that the first case of EVD appeared in the Democratic Republic of the Congo, while 55.1% (n = 109) rated EVD as a highly dangerous illness. On asking about the natural reservoir of EVD, around half of the students (51.5%; n = 102) correctly recognized fruit bats as the natural source. In addition, about 42% (n = 83) indicated that EVD could be contracted via direct contact with blood and body fluids. Less than a quarter of the students (23.7%; n = 47) knew that survivors with PES can transmit the disease to their contacts. Overall, the participants had a poor knowledge score, with only 16.7% (n = 33) answering ≥70% of the knowledge items correctly, resulting in an average score of 5.71 ± 2.25 (range 0-11).

**Table 1:** Participants’ knowledge of Ebola virus disease (n = 198) Data are presented as numbers (%). EVD, Ebola virus disease; PCR, polymerase chain reaction; FDA, Food and Drug Administration; PES, post-Ebola syndrome

Knowledge items	Correct response	n (%)
Country of first EVD case?	Democratic Republic of the Congo	86 (43.4)
EVD severity?	Highly dangerous	109 (55.1)
Causative agent of EVD?	Virus	131 (66.2)
Natural reservoir of EVD?	Fruit bat	102 (51.5)
Mode of transmission of EVD?	Direct contact with blood/ body fluids	83 (41.9)
Incubation period of EVD?	2–21 days	101 (51.0)
Symptoms not included in EVD?	Constipation	41 (20.7)
Confirmatory test for EVD diagnosis?	PCR	69 (34.9)
EVD prevention?	Avoiding contact with blood/body fluids	68 (34.3)
FDA-approved treatment for EVD?	Yes	122 (61.6)
FDA-approved vaccine to prevent EVD?	Yes	119 (60.1)
Can Survivors with PES transmit EVD?	Yes	47 (23.7)

Association between students’ gender and knowledge of EVD

As shown in Table 2, male students were more knowledgeable about the causative agent of EVD (74.2%; n = 69) than female students (59%; n = 62), with a statistically significant difference (p = 0.002). In addition, 67.7% (n = 63) of male students knew that there is a vaccine currently available for EVD, compared with 53.3% (n = 56) of female students (p = 0.03). In response to the item regarding the natural reservoir of EVD, 52.4% (n = 55) of female students answered correctly, compared with 50.5% (n = 47) of male students (p = 0.02). Among male students, the overall mean knowledge score was 6.22 ± 2.13 versus 5.24 ± 2.26 for female students, with a statistically significant difference (p = 0.02).

**Table 2:** Cross-tabulation of students’ gender with knowledge of Ebola virus disease Data are presented as numbers (%). *Statistically significant (p <0.05). EVD, Ebola virus disease; χ2, Pearson’s chi-square; PCR, polymerase chain reaction; FDA, Food and Drug Administration; PES, post-Ebola syndrome

Knowledge items	Correct answer	Males % (n = 93)	Females % (n = 105)	χ^2^	p-value
Source of information about EVD?	Internet	55 (59.1)	47 (44.7)	4.56	0.34
Country of first EVD case?	Democratic Republic of the Congo	45 (48.3)	41 (39.0)	2.27	0.51
EVD severity?	Highly dangerous	60 (64.5)	49 (46.7)	6.83	0.06
Causative agent of EVD?	Virus	69 (74.2)	62 (59.0)	16.77	0.002*
Natural reservoir of EVD?	Fruit bat	47 (50.5)	55 (52.4)	9.41	0.02*
Mode of transmission of EVD?	Direct contact with blood/ body fluids	38 (40.9)	45 (42.9)	5.45	0.14
Incubation period of EVD?	2–21 days	54 (58.1)	47 (44.8)	4.34	0.22
Symptoms not included in EVD?	Constipation	21 (22.5)	20 (19.0)	6.22	0.09
Confirmatory test for EVD?	PCR	34 (36.6)	35 (33.3)	0.73	0.86
EVD prevention?	Avoiding contact with blood/body fluids	31 (33.3)	37 (35.2)	8.21	0.06
FDA-approved treatment for EVD?	Yes	51 (54.8)	71 (67.6)	3.41	0.06
FDA-approved vaccine for EVD?	Yes	63 (67.7)	56 (53.3)	4.27	0.03*
Can Survivors with PES transmit EVD?	Yes	21 (22.6)	26 (24.8)	0.13	0.74

Correlation between students’ academic year and knowledge of EVD

The internet ranked first among the sources of information on EVD for the three academic years. Most fourth-year medical students (95.6%; n = 43) and about 30% (n = 25) of third-year students knew that the first case of Ebola was reported in the Democratic Republic of the Congo; however, 33.3% (n = 23) of second-year students reported China as the country of origin (p = 0.0001). The great majority of fourth-year students were aware of the natural source of EVD (91.2%; n = 41), compared with 44.9% (n = 31) and 35.7% (n = 30) of second- and third-year students, respectively.

When asked about the laboratory diagnosis of EVD, a large proportion of fourth-year students (88.9%; n = 40) and approximately 35% (n = 29) of third-year students recognized polymerase chain reaction (PCR) as the optimal test. In contrast, none of the second-year students was aware of the test of choice (p = 0.0001). The three academic years showed significantly different responses regarding the mode of transmission (p = 0.002) and prevention of EVD (p = 0.001). Furthermore, about two-thirds of fourth-year students (68.9%; n = 31) knew that survivors with PES can spread the virus to others. The overall mean knowledge score for the second-, third-, and fourth-year students was 4.79 ± 1.93, 5.31 ± 2.08, and 7.84 ± 1.58, respectively, with a statistically significant difference (p = 0.0001).

Attitudes of the participants toward EVD

**Figure 1:**
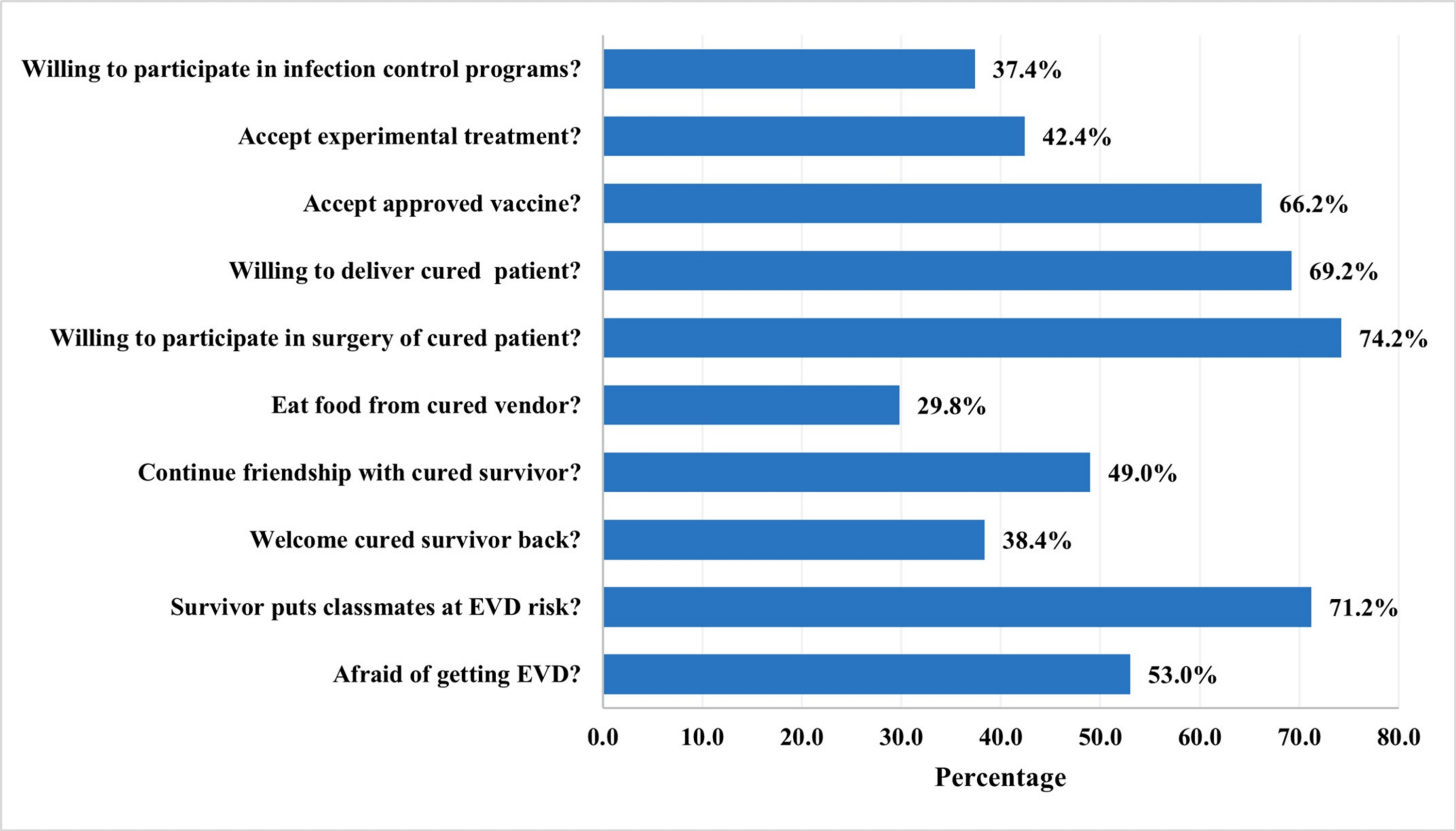
Reported positive attitudes among the study cohort Percentages represent the proportion of students who expressed positive attitudes toward EVD (n = 198). EVD, Ebola virus disease

In general, the students had mixed attitudes. For example, over half (53%; n = 105) reported that they were very afraid of acquiring EVD, while 71.2% (n = 141) thought that there was a greater risk of disease transmission when sharing classes with EVD survivors. In addition, only 38.4% (n = 76) would welcome back into the community a survivor who had been declared cured of EVD. On the other hand, 74.2% (n = 147) of the students expressed willingness to contribute to the surgical interventions for EVD-cured patients, and 69.2% (n = 137) would assist in the childbirth of pregnant women who had survived EVD. The mean attitude score was 4.65 ± 1.78 (range 0-8). Figure 1 highlights the frequency of reported positive attitudes among the participants.

Practices of medical students in relation to EVD

**Figure 2:**
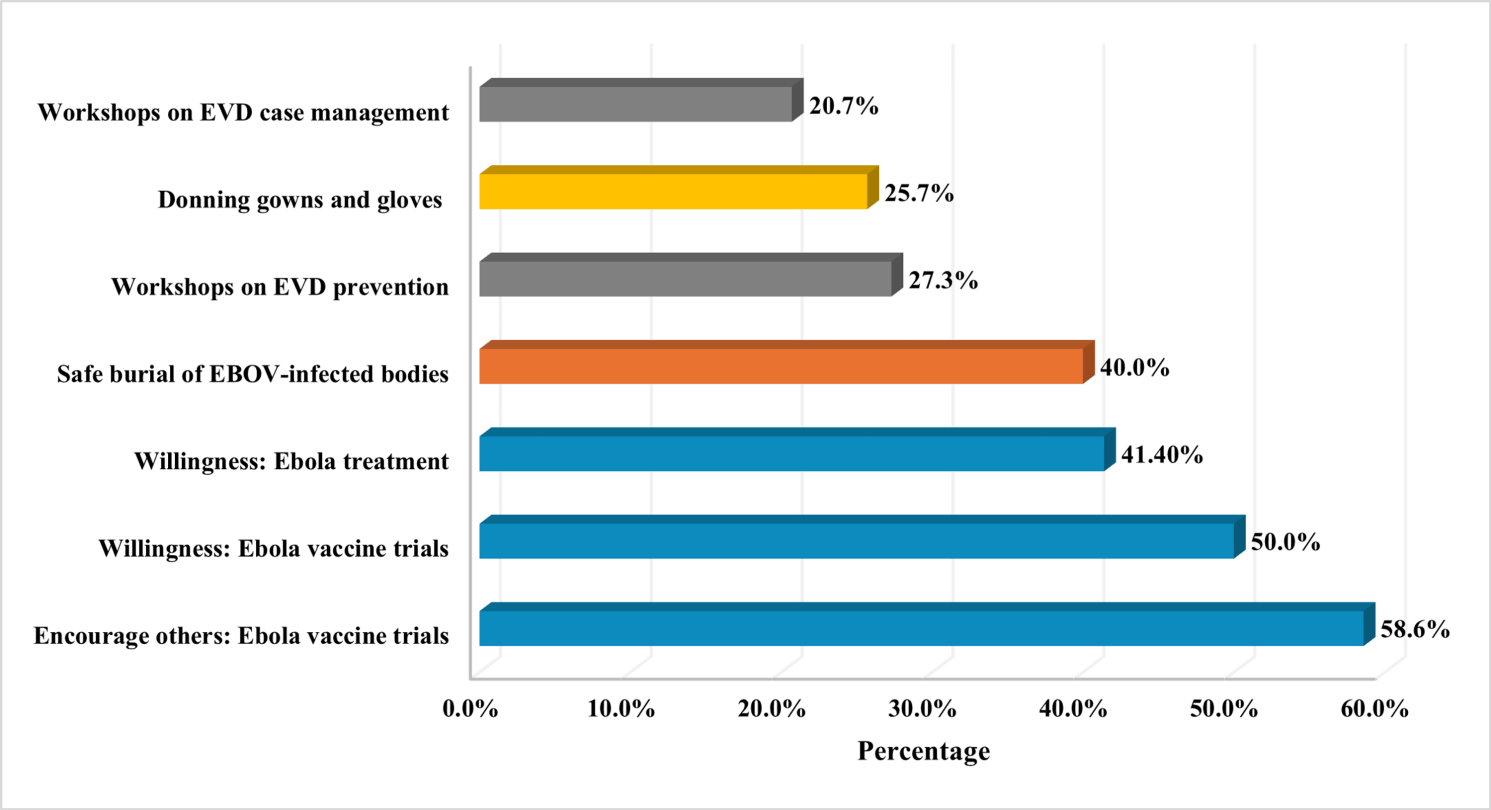
Reported appropriate practices of medical students regarding Ebola virus disease Percentages represent the proportion of students who demonstrated adequate practices (n = 198). EVD, Ebola virus disease; EBOV, Ebola virus

A significant majority of the students (91.4%) exhibited inadequate practices. Most of the students reported not having participated in workshops on EVD prevention (72.7%; n = 144) or case management (79.3%; n = 157). Only 41.4% (n = 116) of the participants indicated willingness to work in Ebola treatment/isolation centers during outbreaks. Half of the cohort (50%; n = 99) expressed willingness to volunteer in Ebola vaccine trials, and 58.6% (n = 116) indicated that they would encourage their friends and family members to participate in such trials. Of concern, only 25.7% (n = 51) of the students underscored the importance of using gowns and gloves to protect HCWs against EVD. When asked about measures for handling Ebola cadavers, 60% (n = 119) of students considered traditional burials sufficient, while 40% (n = 79) acknowledged the importance of safe burials. The average practices score was 2.80 ± 1.29 (range 0-6). Figure 2 presents the frequency of appropriate practices of the study cohort regarding EVD.

Contributing factors and predictors of good knowledge of EVD

Factors potentially affecting EVD knowledge were analyzed using univariate regression analysis, as shown in Table 3. Among the variables analyzed, academic year (p <0.001) and age (p = 0.03) were significantly associated with good knowledge of EVD. A subsequent multivariate regression model indicated that the academic year was the only independent predictor of good knowledge of EVD (OR = 8.35, 95% CI: 3.27-21.33, p <0.001).

**Table 3:** Factors associated with good knowledge of Ebola virus disease Data are presented as numbers (%). *Statistically significant (p <0.05). EVD, Ebola virus disease; χ2, Pearson’s chi-square

Parameters	Good knowledge (n = 33)	Poor knowledge (n = 165)	χ^2^	p-value
Age (years)				
<20	4 (12.1%)	48 (29.1%)	4.09	0.03*
≥20	29 (87.9%)	117 (70.9%)		
Gender				
Male	20 (60.6%)	73 (44.2%)	2.95	0.08
Female	13 (39.4%)	92 (55.8%)		
Nationality				
Saudi	32 (97.0%)	137 (83.0%)	4.27	0.06
Non-Saudi	1 (3.0%)	28 (17.0%)		
Academic year				
Second	4 (12.1%)	65 (39.4%)	38.11	<0.001*
Third	8 (24.2%)	76 (46.1%)		
Fourth	21 (63.7%)	24 (14.5%)		
Source of information about EVD				
Internet	20 (60.6%)	82 (49.7%)	6.32	0.17
Lectures	2 (6.0%)	35 (21.2%)		
Television	5 (15.2%)	21 (12.7%)		
Colleague	5 (15.2%)	14 (8.5%)		
Newspaper	1 (3.0%)	13 (7.9%)		

Correlation between knowledge, attitude, and practice domains

According to Pearson’s correlation analysis, a weak positive correlation was identified between knowledge and attitudes (r = 0.17). The correlation was statistically significant (p = 0.012), meaning that higher levels of knowledge were associated with more favorable attitudes toward EVD. On the other hand, the correlation between knowledge and practices was negligible (r = 0.003, p = 0.96). A moderate positive correlation was found between attitudes and practices (r = 0.35). This correlation was statistically significant (p <0.001), indicating that more positive attitudes were associated with better practices.

## Discussion

The KAP surveys/questionnaires are widely used tools for measuring the level of awareness and preventive practices for a certain public health issue, including EVD. Our study highlights considerable gaps in the understanding of EVD that warrant targeted educational efforts. For example, 56.6% (n = 112) of the medical students were not aware of the index case of Ebola in the Democratic Republic of the Congo, which is consistent with recent Ugandan data showing suboptimal EVD knowledge among medical students [15]. Furthermore, only about 42% (n = 83) of the responding students were mindful of the mode of transmission of EVD. Consistent with our results, a recent hospital-based survey among frontline HCWs reported substantial gaps in the knowledge of EVD transmission, with only 34% of participants knowing that contact with contaminated clothing is a potential route of transmission [16]. Poor knowledge of how an infectious disease can be acquired is a key predisposing factor for the rapid spread within the community, as HCWs may fail to adhere to standard infection prevention and control precautions, thereby promoting further disease dissemination.

In our study, 65.1% (n = 129) of medical students were unaware of the optimum diagnostic technique for confirming suspected EVD cases. This gap underlines the urgent need to integrate research projects addressing emerging and re-emerging viral threats into medical curricula, to enhance students’ understanding of the scope of this problem and the importance of prompt diagnosis. Compared to our findings, 62.7% of the participating HCWs in a multicenter nationwide survey from Somalia demonstrated adequate knowledge regarding laboratory diagnosis of EVD [17]. This discrepancy may be attributed to differences in study participants and setting, as the Somalian study recruited physicians, pharmacists, nurses, midwives, and laboratory technicians, whereas our cohort included undergraduate students with limited clinical experience [17].

Only a small proportion of our students (23.7%; n = 47) were aware of the potential transmissibility of EBOV from survivors who suffer from PES. Recently, persistence of EBOV in the central nervous system has been reported, including survivors experiencing cerebral recrudescence. It is suggested that Ebola virions can persist in immunologically privileged tissues, including the eye, brain, testes, placenta, and amniotic fluid, posing a risk of disease transmission, recrudescence, and re-emergence of future outbreaks [18]. These findings underscore the need for robust research initiatives and proactive preparedness to mitigate the risk of EVD resurgence.

While satisfactory knowledge is the backbone for building up positive attitudes and encouraging effective practices, our questionnaire demonstrated an overall poor knowledge score (5.71 ± 2.25), with only 16.7% (n = 33) of students answering ≥70% of the knowledge questions correctly. This deficiency points to the necessity for targeted Ebola training programs for both undergraduate and postgraduate candidates in our institution. Compared with our students, HCWs in a Nigerian study demonstrated greater awareness of EVD [19]. This disagreement is largely due to differences in inclusion criteria, as the Nigerian study included medical practitioners, nurses, and other HCWs with previous exposure to outbreak-related training, whereas our study was limited to undergraduate students.

Compared with female students, male students were generally more informed about the causative agent of EVD (p = 0.002) and the availability of an FDA-approved vaccine against the disease (p = 0.03). By contrast, female students showed a better understanding of the natural reservoir of EVD than male students (p = 0.02). Similar gender-related differences in knowledge were reported in a recent survey from Rwanda [20]. In general, knowledge of EVD increased with academic level, with fourth-year students showing higher levels of knowledge than their second- and third-year peers. Consistent with our results, Cénat et al. found higher education attainment to be associated with better knowledge of EVD [21]. These observations reflect that clinical exposure in later academic years may improve understanding of EVD, underscoring the need for developing tailored educational interventions for junior students.

The reporting of unfavorable attitudes toward EVD in our cohort was not surprising. For instance, most of the students indicated that they would not welcome contact with EVD survivors, either in the community (61.6%; n = 122) or during classroom settings (71.2%; n = 141). This finding is concerning, as such fears can undermine public health efforts and complicate the reintegration of survivors into their communities. Our results conform to a previous study from Sierra Leone, where EVD survivors encountered stigma upon returning to their home communities [22].

Of interest, 74.2% (n = 147) of students were willing to participate in surgical interventions for EVD survivors, while 69.2% (n = 137) would assist in childbirth for pregnant women who had recovered from EVD. This result aligns with a study conducted in the United States, in which 87.5% of registered nurses expressed a professional responsibility to provide care for patients with EVD [23]. Conversely, 86.8% of emergency HCWs in Uganda expressed reluctance to be in close contact with patients suspected of having or confirmed to have EVD, and 40.4% were anxious about working in a hospital with an EVD treatment unit [24]. These findings highlight the critical need to protect HCWs while caring for patients with contagious diseases by providing a safe hospital environment and implementing effective infection control measures.

Our study uncovered substantial gaps in practice regarding EVD, with most students having not participated in workshops on how to manage infected cases (79.3%; n = 157). The lack of hands-on training and practical skills may hinder the ability of future HCWs to respond effectively and confidently during outbreak situations. This finding is consistent with a KAP survey conducted among Sudanese HCWs [25]. Approximately 59% (n = 82) of our students were unwilling to work in Ebola isolation units/treatment centers. This hesitancy may stem from misconceptions about the current availability of an approved Ebola vaccine and therapy that should be addressed in focused educational initiatives. Notably, a recent study from Uganda reported a widespread reluctance among HCWs to engage in the management of Ebola-related emergencies [26].

Importantly, half of the medical students (n = 99) reported readiness to volunteer for Ebola vaccine trials. By contrast, data from the Democratic Republic of the Congo showed very high overall Ebola vaccine uptake (99%), although first-offer acceptance was lower [27]. Concerns about trial procedures, mistrust, and participant experience were the most noteworthy barriers in recent Ebola vaccine-trial settings [28]. It is therefore necessary to give trial participants correct and comprehensive data, not only from an ethical perspective, but also to foster trust and active engagement in such trials.

A significant proportion of our students (74.3%; n = 147) underestimated the protective role of personal protective equipment (PPE), especially the use of gowns and gloves when caring for EBOV-infected patients. This gap probably arises from insufficient exposure to practical scenarios, including outbreak simulations and case management. Accordingly, it is necessary to integrate skills-based training on standard and transmission-based infection control precautions, including the proper donning and doffing of PPE. Similarly, a Ugandan study found that 78.5% of HCWs were unable to properly don PPE and 68.6% could not correctly doff PPE [26]. Though traditional burial practices represent a major driver for EVD transmission, only 40% (n = 79) of our students correctly recognized the importance of safe disposal and burial of EBOV-infected cadavers. Recently, a study conducted in the Democratic Republic of the Congo during an EVD outbreak showed that burial workers encountered significant community resistance due to misconceptions about safe burial procedures [29].

Given the noteworthy gaps in EVD-related knowledge and practices, along with negative attitudes among our students, we further investigated the correlations between these three domains. Our results demonstrated a weak positive correlation between knowledge and attitude (p = 0.012), whereas a negligible correlation was identified between knowledge and practices (p = 0.96). Similarly, Kunna et al. found that knowledge contributes to shaping attitudes and practices among Sudanese HCWs, although the strength of these relationships is variable [25]. Differences in the study setting and population are likely explanations for this inconsistency. Taken together, these results show that improving knowledge alone might not be enough to guarantee appropriate practices. Thus, in-depth interventions that also address attitudes, behavioral factors, and practice-based learning are warranted.

Study limitations

This study has several limitations that warrant careful consideration. First, the students were exclusively recruited from a single institution in Saudi Arabia, which may restrict the generalizability of our results to the broader population. Second, the study relied on a self-administered questionnaire, which may introduce self-reporting bias and social desirability bias, potentially affecting the accuracy of the responses. Furthermore, the distribution of the questionnaire via WhatsApp (Meta Platforms, Inc.) may have introduced selection bias, as only students with access to and engagement with the platform were able to participate, which may limit the representativeness of the sample. Third, the study did not include supplementary queries to understand the rationale behind the participants’ answers. Fourth, although 198 students participated in the study, the overall response rate was 44%, which may introduce non-response bias, as the views of non-respondents may differ from those who participated. Finally, the cross-sectional design of the study provides responses at a single time point, precluding the analysis of changes in knowledge, attitudes, or practices across time. These limitations should be considered when interpreting the findings and addressed in future research.

## Conclusions

This study highlights gaps in the knowledge, attitudes, and practices of medical students at FCMS regarding EVD. While the students have a basic understanding of the disease, there is much room for improvement, especially in understanding transmission, prevention, and survivor stigmatization. These findings suggest a need for tailored educational programs and enhanced hands-on training opportunities for medical students. Addressing these shortcomings may help better prepare future HCWs to care for individual patients and support public health responses during potential EVD outbreaks.

## Appendices

Questionnaire used for assessment of knowledge, attitudes, and practices regarding Ebola virus disease (including demographic data and information sources):

https://docs.google.com/spreadsheets/d/1z5Ox_C2QoFDEOevwd822CMkOw3Apm43u/edit?usp=sharing&ouid=112790397093314458241&rtpof=true&sd=true
